# Supplementary material for: Resolving haplotype variation and complex genetic architecture in the human immunoglobulin kappa chain locus in individuals of diverse ancestry
Source: Genes Immun. 2024 Jun 6;25(4):297–306. doi: 10.1038/s41435-024-00279-2 (PMC11327106; doi:10.1038/s41435-024-00279-2)
Supplement: Supplementary file 1 — Supplementary figures [file 41435_2024_279_MOESM1_ESM.pdf]

# Resolving haplotype variation and complex genetic architecture in the human immunoglobulin kappa chain locus in individuals of diverse ancestry

## Supplementary Figures

Eric Engelbrecht, Oscar L. Rodriguez, Kaitlyn Shields, Steven Schultze, David Tieri, Uddalok Jana, Gur Yaari, William Lees, Melissa L. Smith*, Corey T. Watson*

# Figure S1

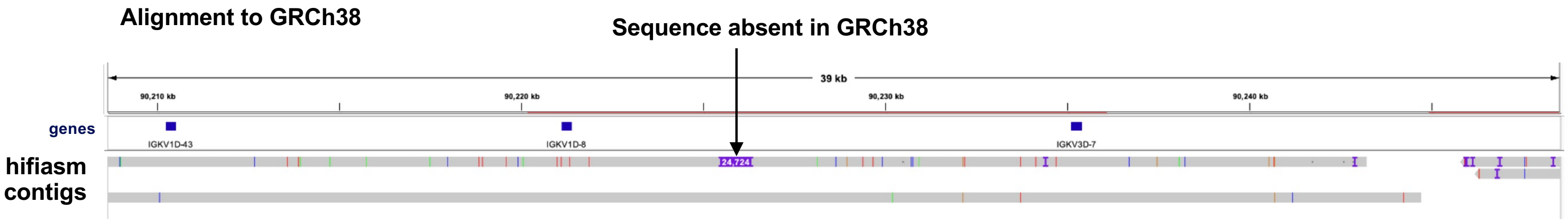


**Supplementary Figure 1.**
IGV screenshot of hifiasm-generated contigs from an AFR sample mapped to the GRCh38 genome assembly. One of the contigs (one haplotype) has a ~24.7 Kbp insertion between IGKV1D-8 and IGKV3D-7.

# Figure S2

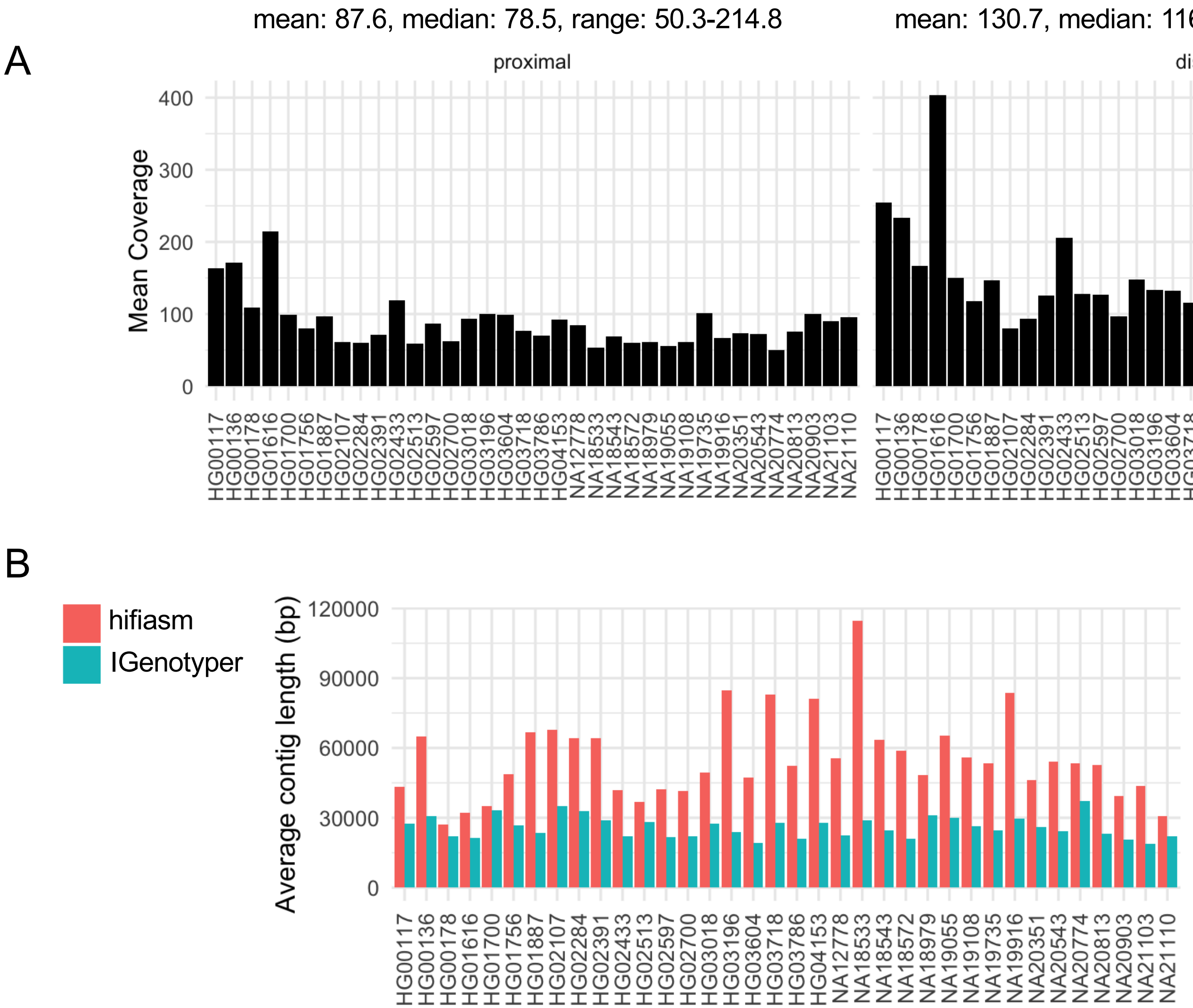


| Average contig length (bp) | | |
|---|---|---|
| | hifiasm | IGenotyper |
| mean | 55400.7 | 25948.1 |
| median | 52973.2 | 25332.4 |
| min | 27230.0 | 18928.7 |
| max | 114630.7 | 37317.6 |

**Supplementary Figure 2.**
**(A)** HiFi read coverage across the IGK locus for all samples in the cohort. Reads were mapped to our custom reference. **(B)** Average length of contigs mapped to the IGK locus generated by hifiasm and IGenotyper for each sample.

Figure S3

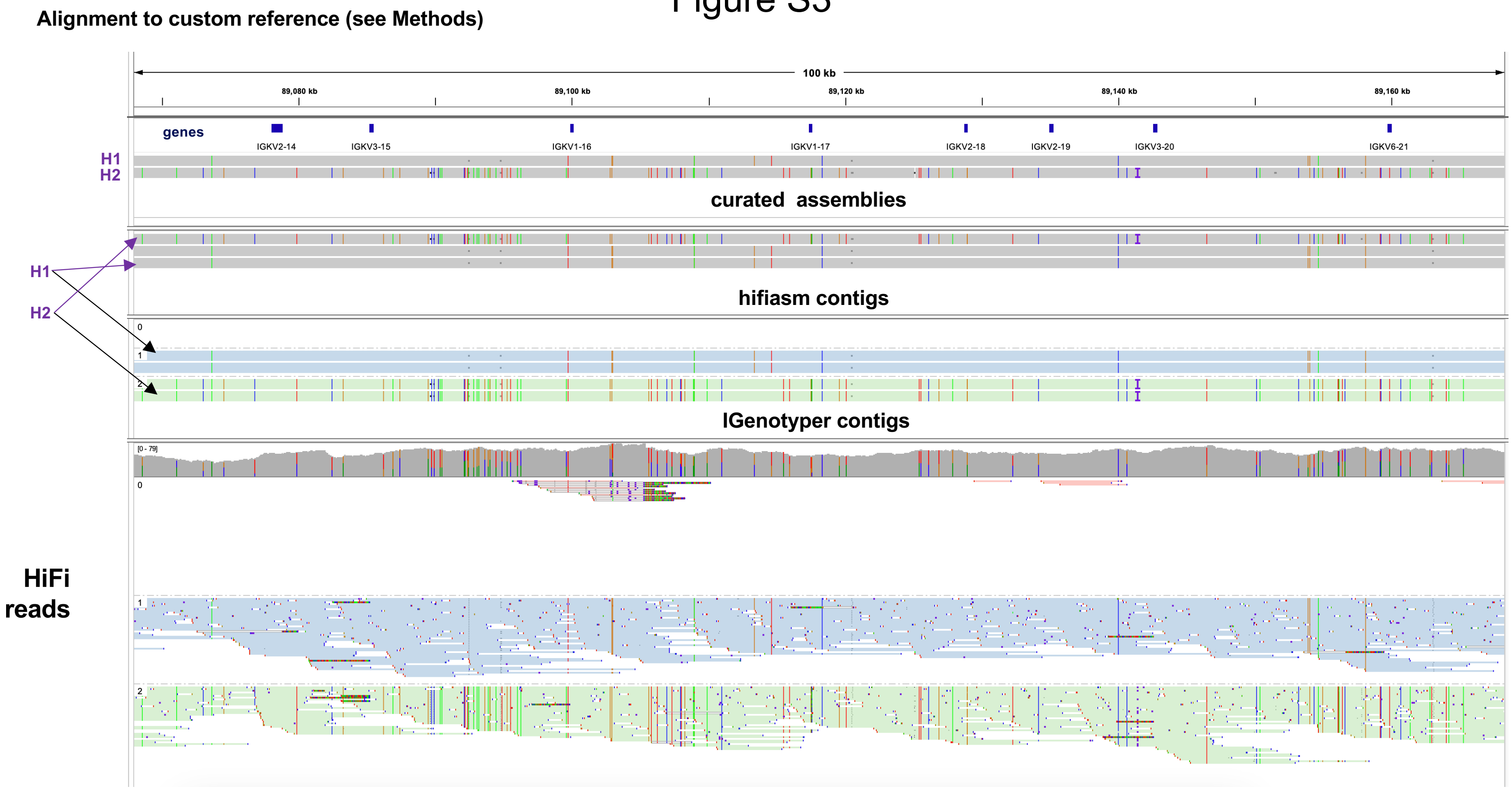


**Supplementary Figure 3.**
IGV screenshot of 100 Kbp in the IGK proximal region with curated assemblies, phased HiFi reads as well as phased contigs generated by IGenotyper and hifiasm aligned to our custom reference. Purple arrows indicate haplotype ("H1" and "H2") contigs selected for curated assembly.

Sample: NA19055

# Figure S4

Sample: NA19055

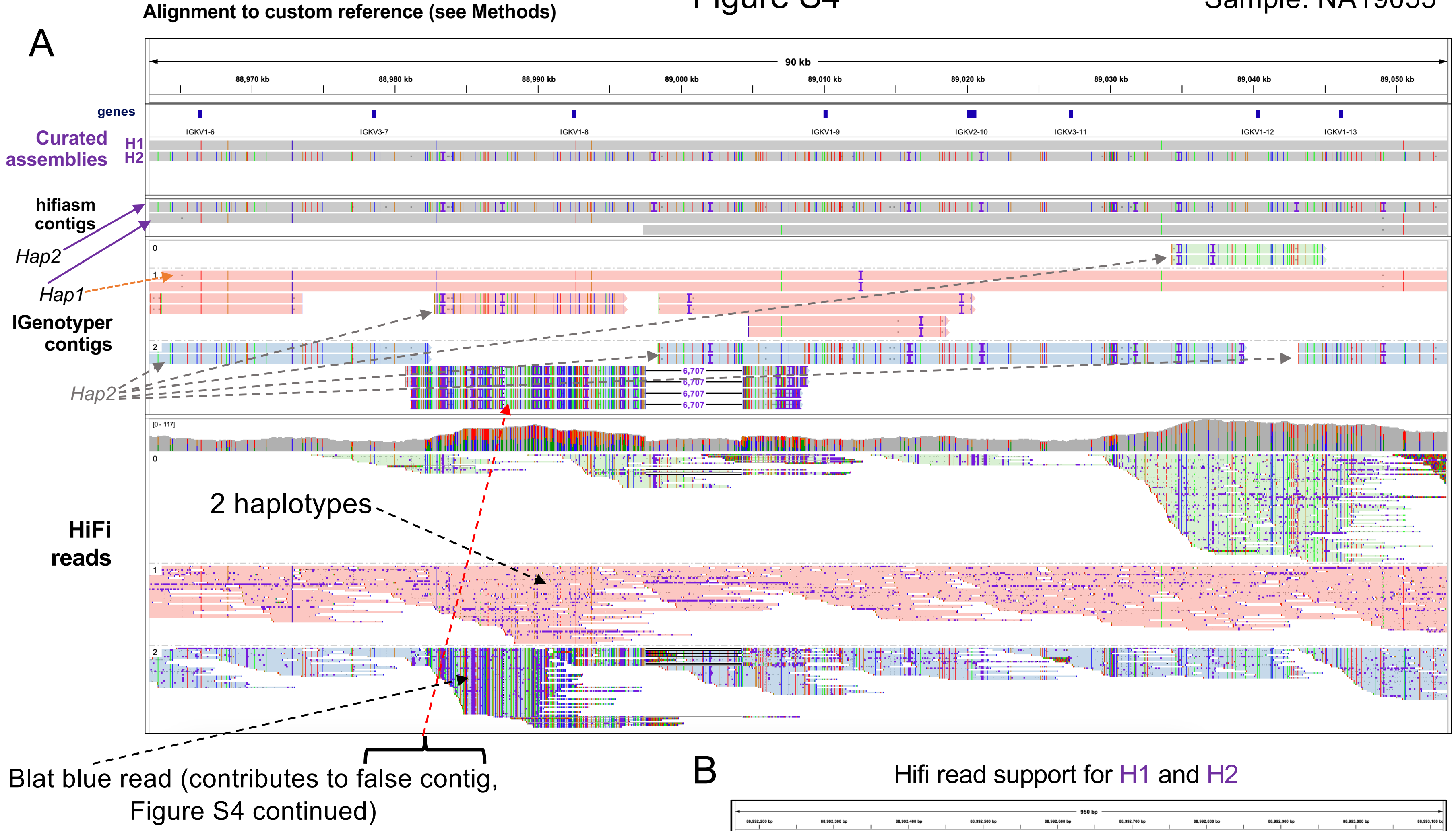


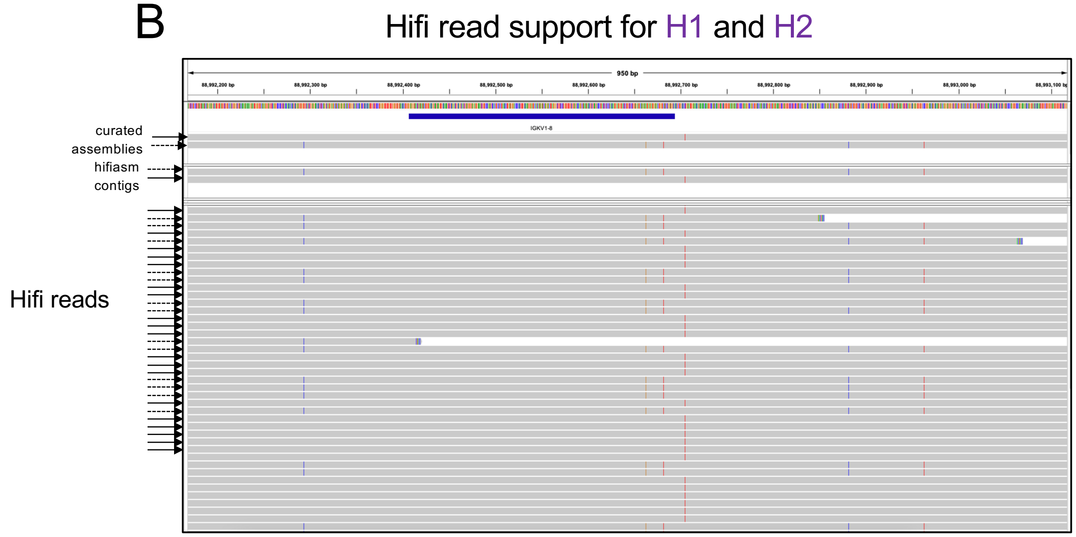


**Supplementary Figure 4.**
**(A)** IGV screenshot showing curated assemblies (H1 and H2), hifiasm contigs, IGenotyper contigs, and HiFi reads aligned to our custom reference. Purple arrows indicate contigs selected for curated assemblies. An IGenotyper contig (orange arrow) congruent with the "Hap1" contig generated by hifiasm (purple arrow). The "Hap2" contig generated by hifiasm (purple arrow) is identified as discontiguous contigs generated by IGenotyper (gray arrows). The red arrow indicates a group of assemblies that likely result from mismapped reads. The black arrow indicates a read that was queried against the GRCh38 genome (continued). **(B)** IGV screenshot of curated assemblies, hifiasm contigs, and HiFi reads, zoomed in to a region containing IGKV1-8 in panel **(A)**. Two SNP patterns in HiFi reads (dashed and solid arrows) are consistent with hifiasm contigs and curated assemblies.

C

**(C)** IGV screenshot showing the aforementioned read, which was queried against the GRCh38 genome using BLAT (UCSC Genome Browser). The best match was to the IGK distal region, between IGKV1D-8 and IGKV1D-7, and the second-best match was to the IGK proximal region, between IGKV3-7 and IGKV1-8.

**Alignment to custom reference (see Methods)**

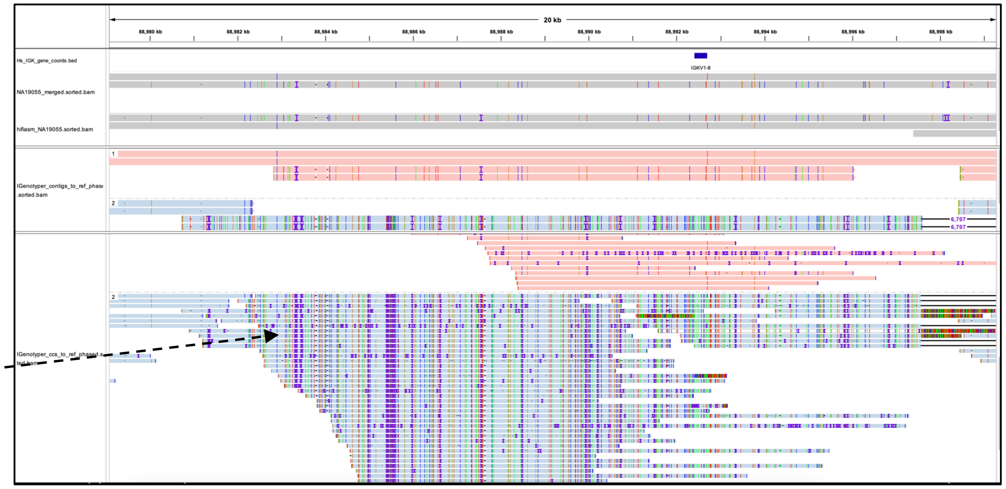

Blat read against hg38

## Blat result

| | ACTIONS | QUERY | SCORE | START | END | QSIZE | IDENTITY | CHROM | STRAND | START | END | SPAN |
|---|---|---|---|---|---|---|---|---|---|---|---|---|
| | browser details | YourSeq | 8814 | 7 | 8828 | 8833 | 100.0% | chr2_KI270894v1_alt | - | 4128 | 37675 | 33548 |
| 1 | browser details | YourSeq | 8800 | 7 | 8828 | 8833 | 99.9% | chr2 | - | 90222464 | 90231291 | 8828 |
| | browser details | YourSeq | 8373 | 7 | 8828 | 8833 | 97.8% | chr2_KQ031384v1_fix | + | 91337 | 100140 | 8804 |
| 2 | browser details | YourSeq | 8373 | 7 | 8828 | 8833 | 97.8% | chr2 | + | 88982511 | 88991314 | 8804 |

1

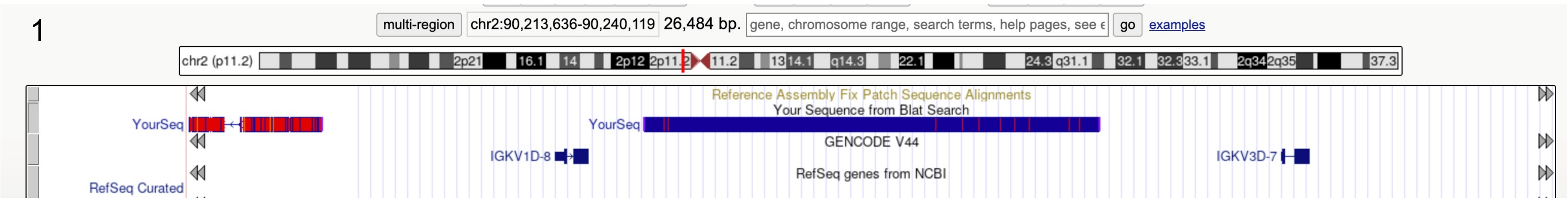


2

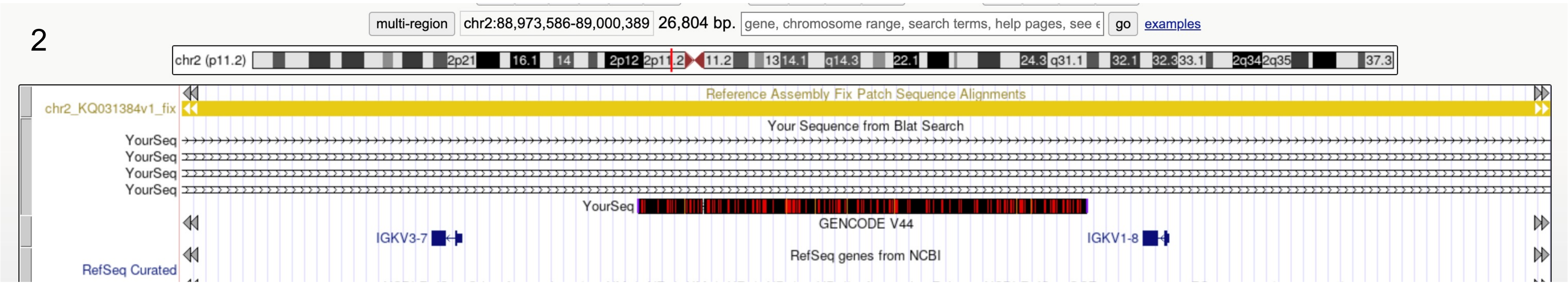


Sample: NA19055

# Figure S5

Sample: HG03196

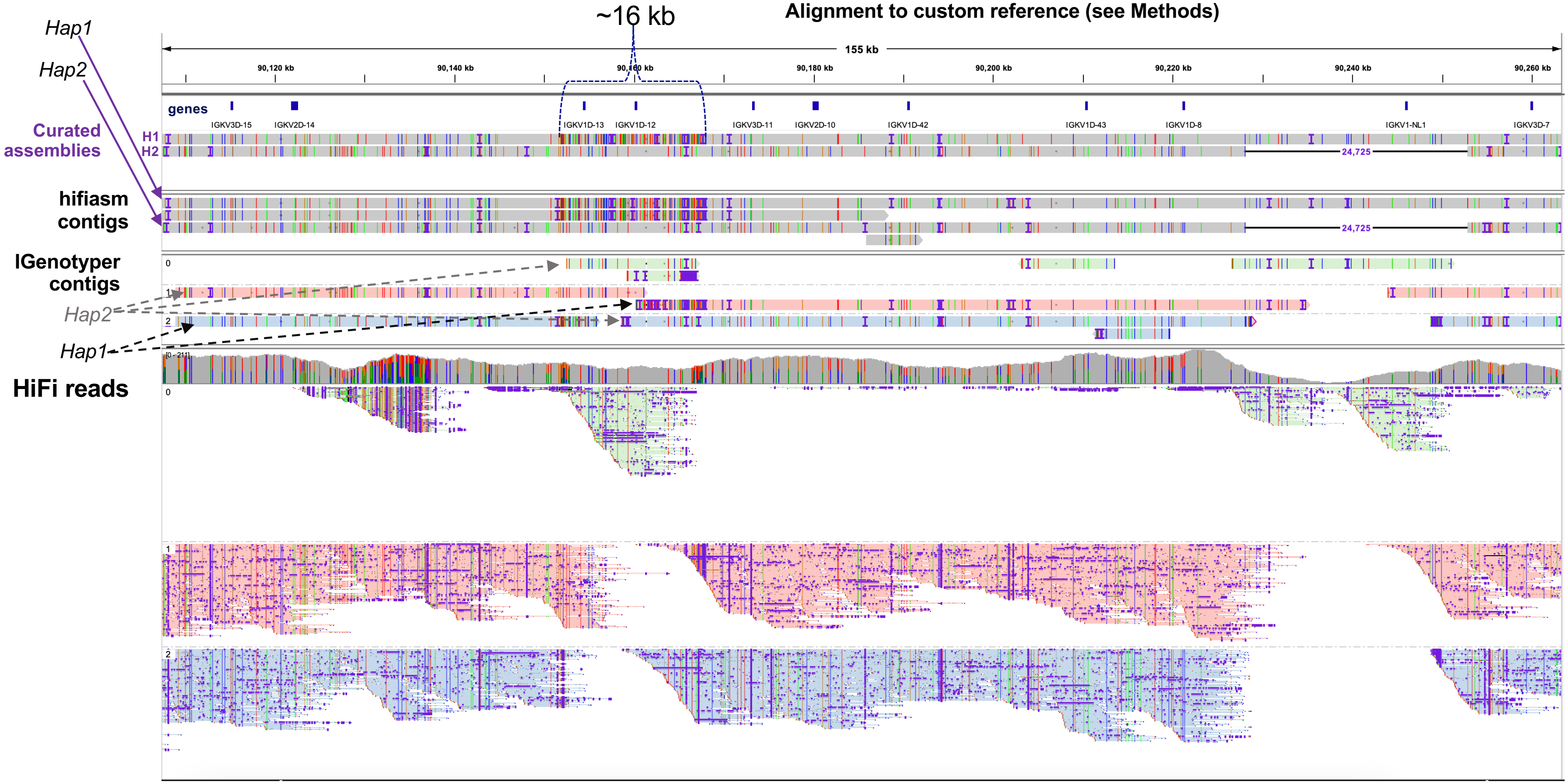


**Supplementary Figure 5.**
IGV screenshot of the IGK distal region spanning IGKV3D-15 to IGKV3D-7, which includes the IGKV1-13/IGKV1D-13; IGKV1-12/IGKV1D-12 gene conversion (~16 Kbp region) and structural variant that includes the gene IGKV1-NL1. Shown are curated assemblies (H1 and H2), hifiasm contigs, IGenotyper contigs, and HiFi reads aligned to our custom GRCh38 reference. Purple arrows indicate hifiasm contigs selected for curated assemblies; “Hap1” (H1) includes the gene conversion event. IGenotyper-generated contigs corresponding to “Hap1” and “Hap2” are fragmented across multiple contigs; hifiasm generated uninterrupted contigs for “Hap1” and “Hap2" in this region.

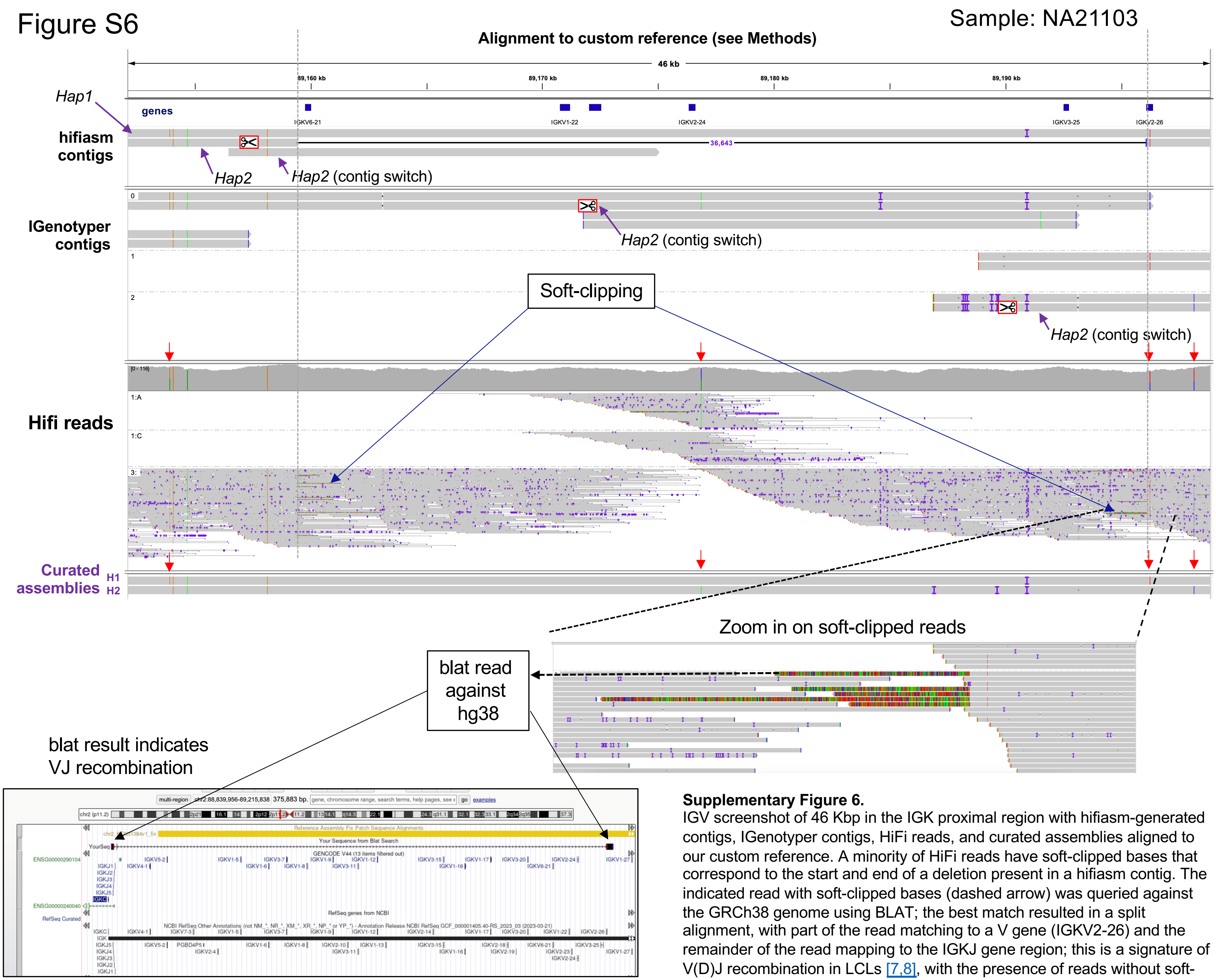


**Supplementary Figure 6.**
IGV screenshot of 46 Kbp in the IGK proximal region with hifiasm-generated contigs, IGenotyper contigs, HiFi reads, and curated assemblies aligned to our custom reference. A minority of HiFi reads have soft-clipped bases that correspond to the start and end of a deletion present in a hifiasm contig. The indicated read with soft-clipped bases (dashed arrow) was queried against the GRCh38 genome using BLAT; the best match resulted in a split alignment, with part of the read matching to a V gene (IGKV2-26) and the remainder of the read mapping to the IGKJ gene region; this is a signature of V(D)J recombination in LCLs [7,8], with the presence of reads without soft-clipping explained by cell line polyclonality [69].

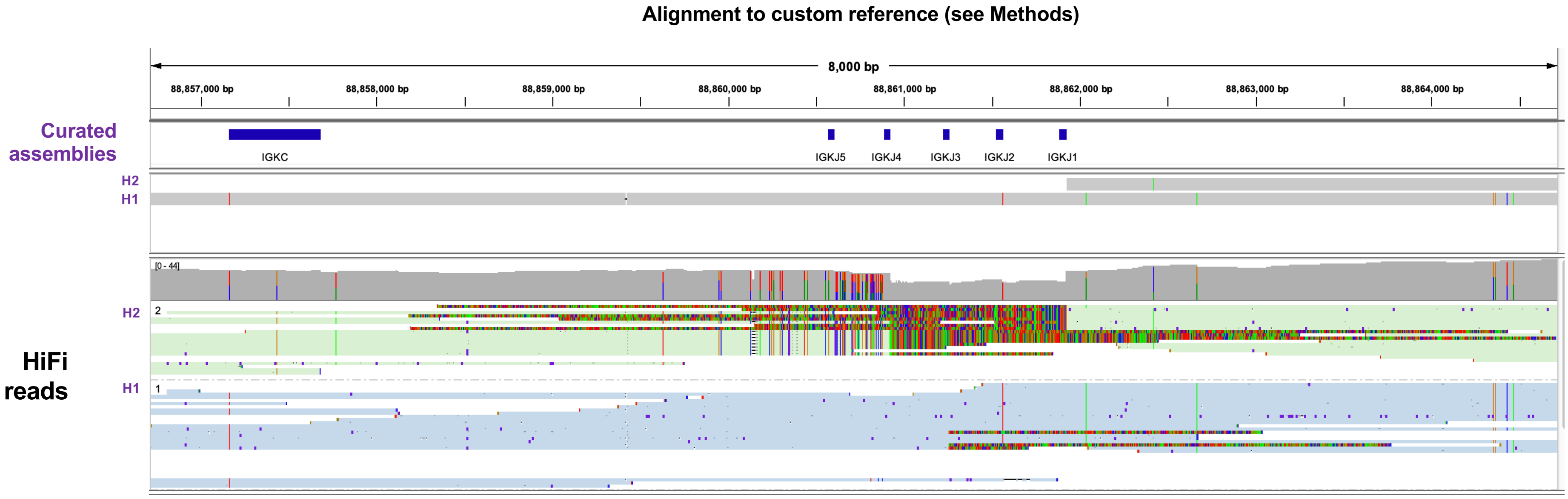


**Supplementary Figure 7.**
Example of a sample for which V(D)J recombination prohibited allele assignments for one haplotype, indicated as “H2”, for the genes IGKC and IGKJ1-5. Assemblies of the other haplotype, “H1”, were included in the analysis because reads corresponding to this haplotype lacked soft-clipping.

Sample: HG02107

# Figure S8

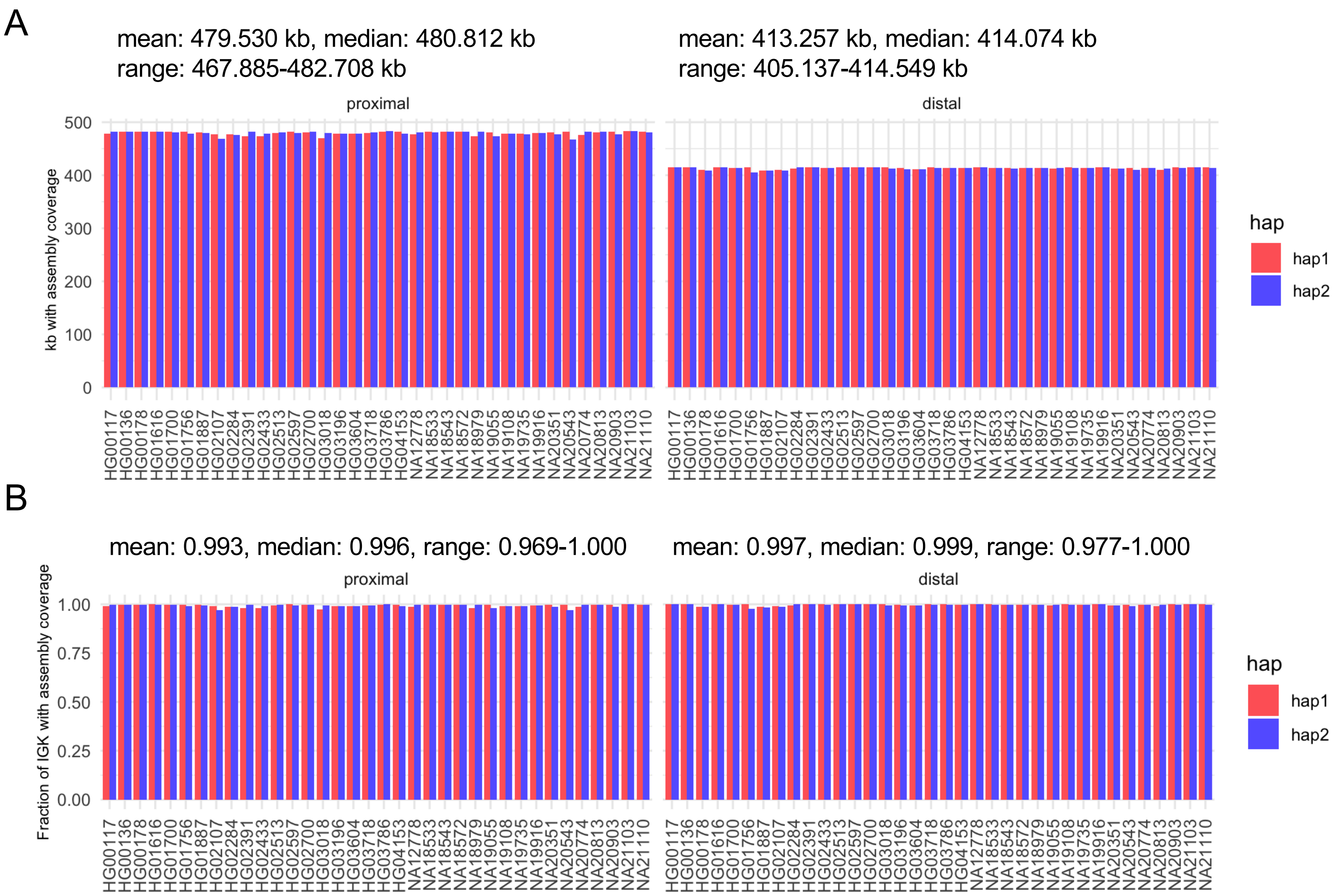


**Supplementary Figure 8.**
**(A)** Number of bases in the IGK locus of our custom reference with curated assembly coverage.
**(B)** Fraction of IGK proximal and distal regions of our custom reference with curated assembly coverage.

# Figure S9

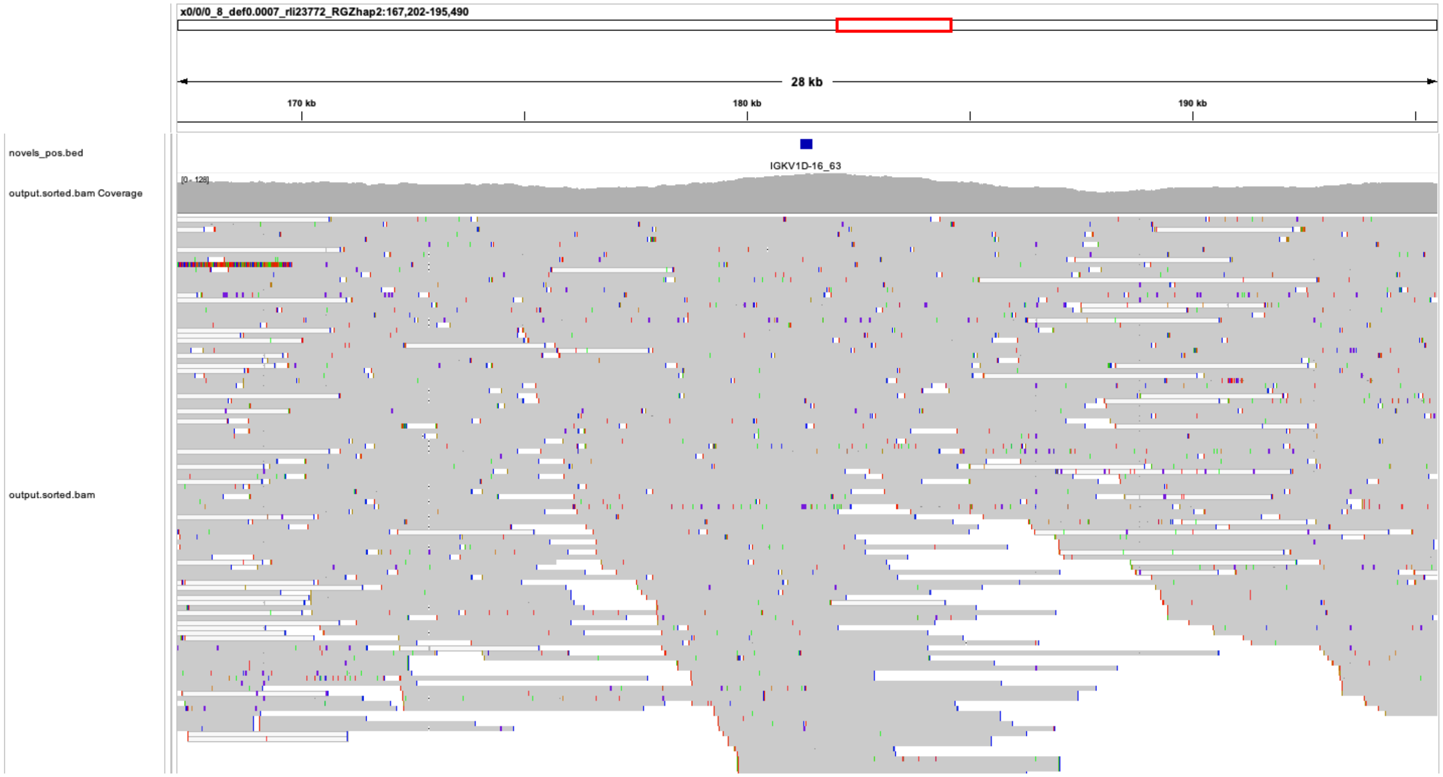


**Supplementary Figure 9.**
IGV screenshots of novel alleles from the sample HG01887 aligned to the individual's IGK-personalized reference (see **Materials and Methods**). Also shown are HiFi reads aligned to the individual's IGK-personalized reference. HiFi reads support (i.e. do not show mismatches with) the contigs selected for diploid IGK assembly. Quantitative assessments of read support for novel alleles are presented in **Table S3**.

Sample: HG01887

# Figure S9
(continued)

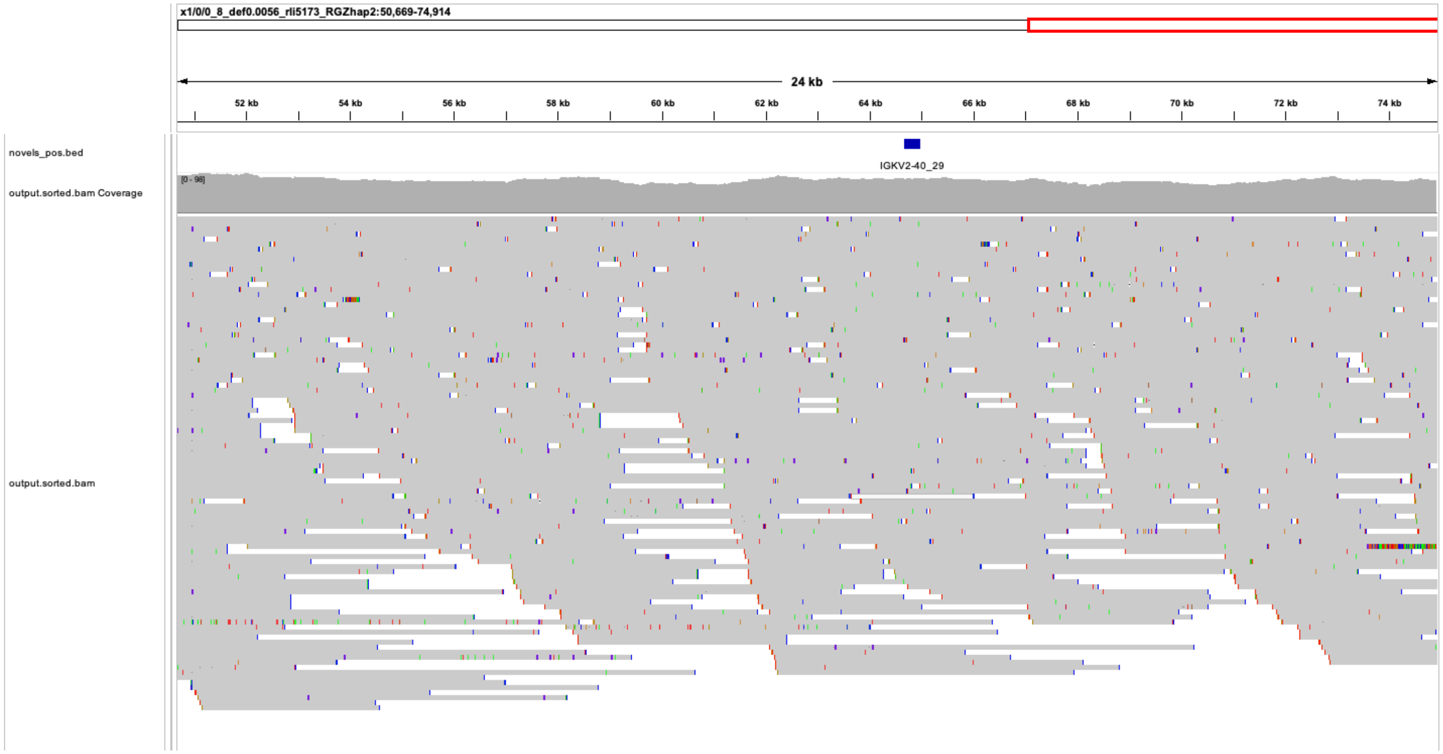


Sample: HG01887

Figure S9
(continued)

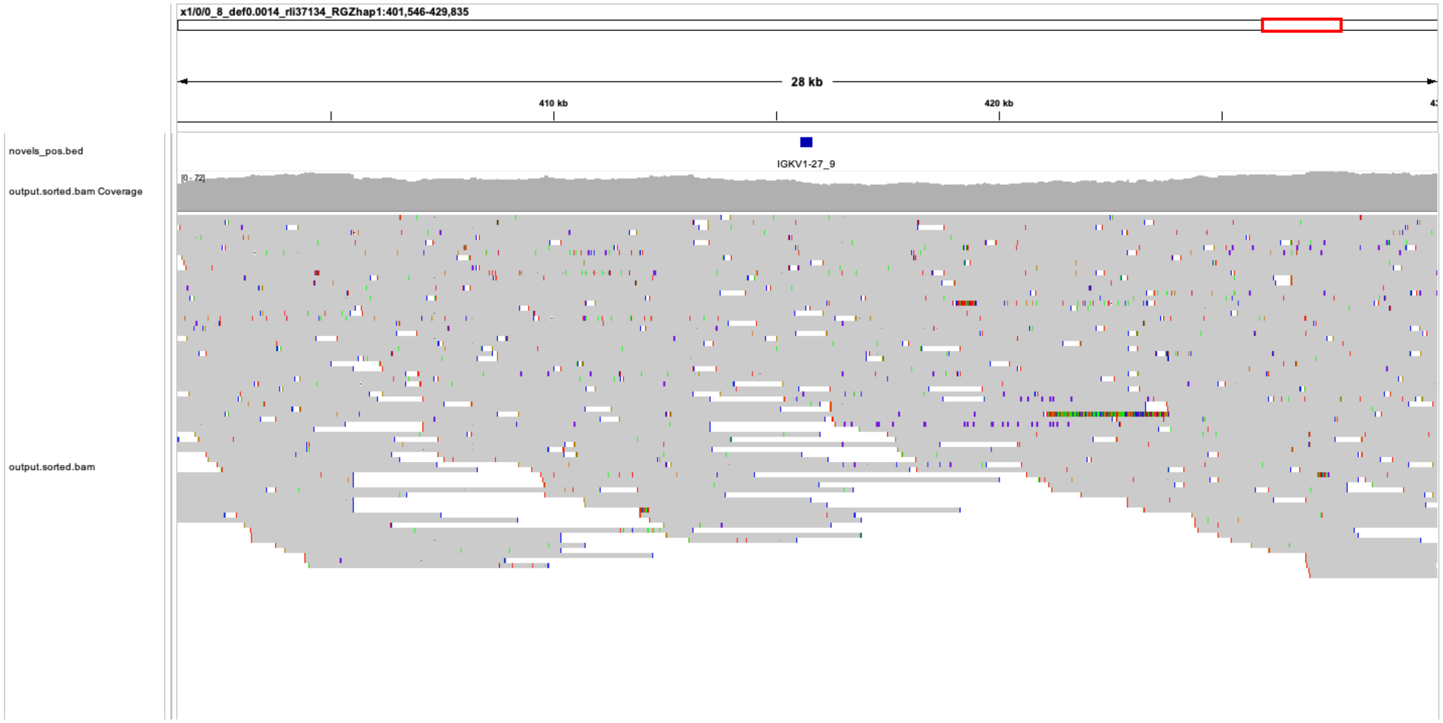


Sample: HG01887

# Figure S9
(continued)

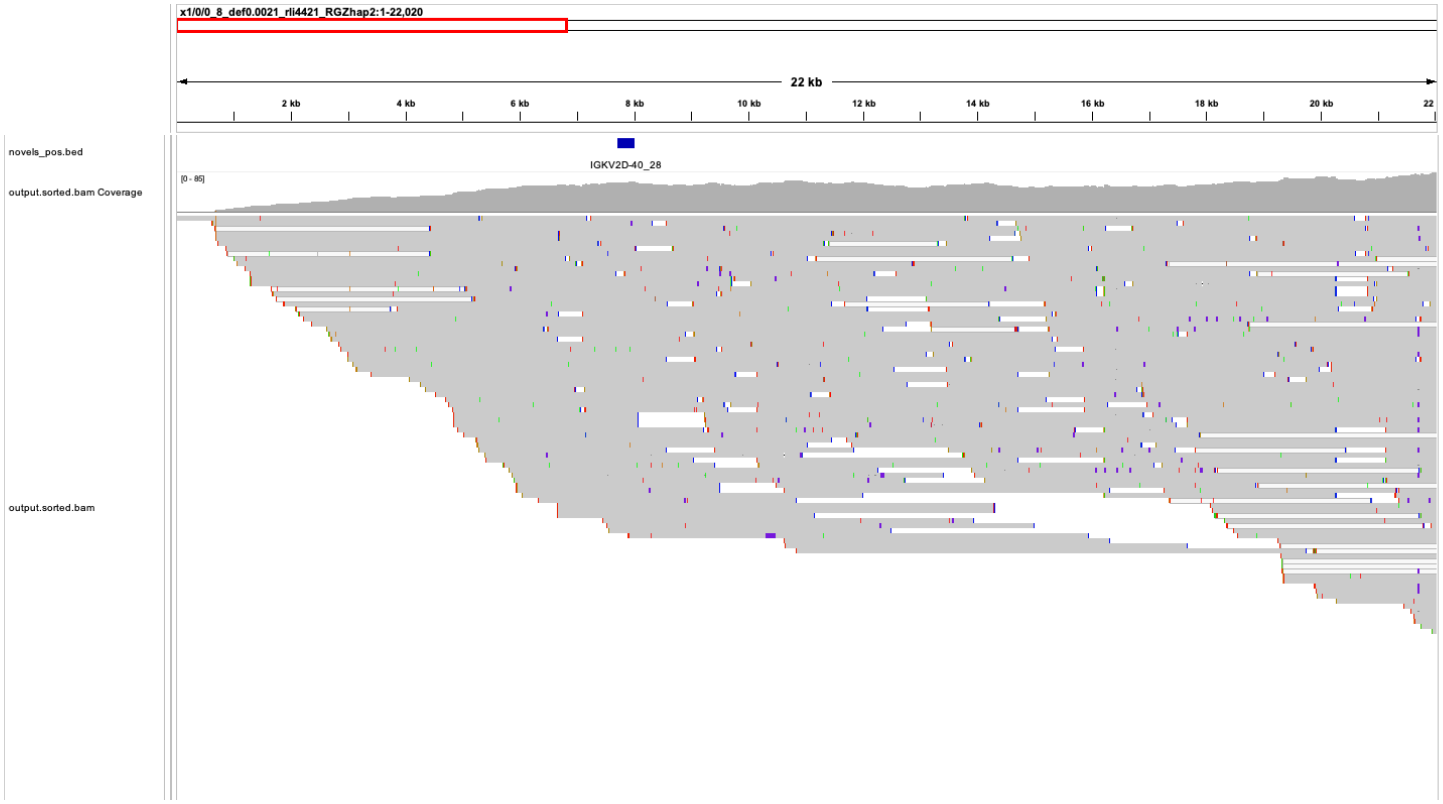


Sample: HG01887

# Figure S9
(continued)

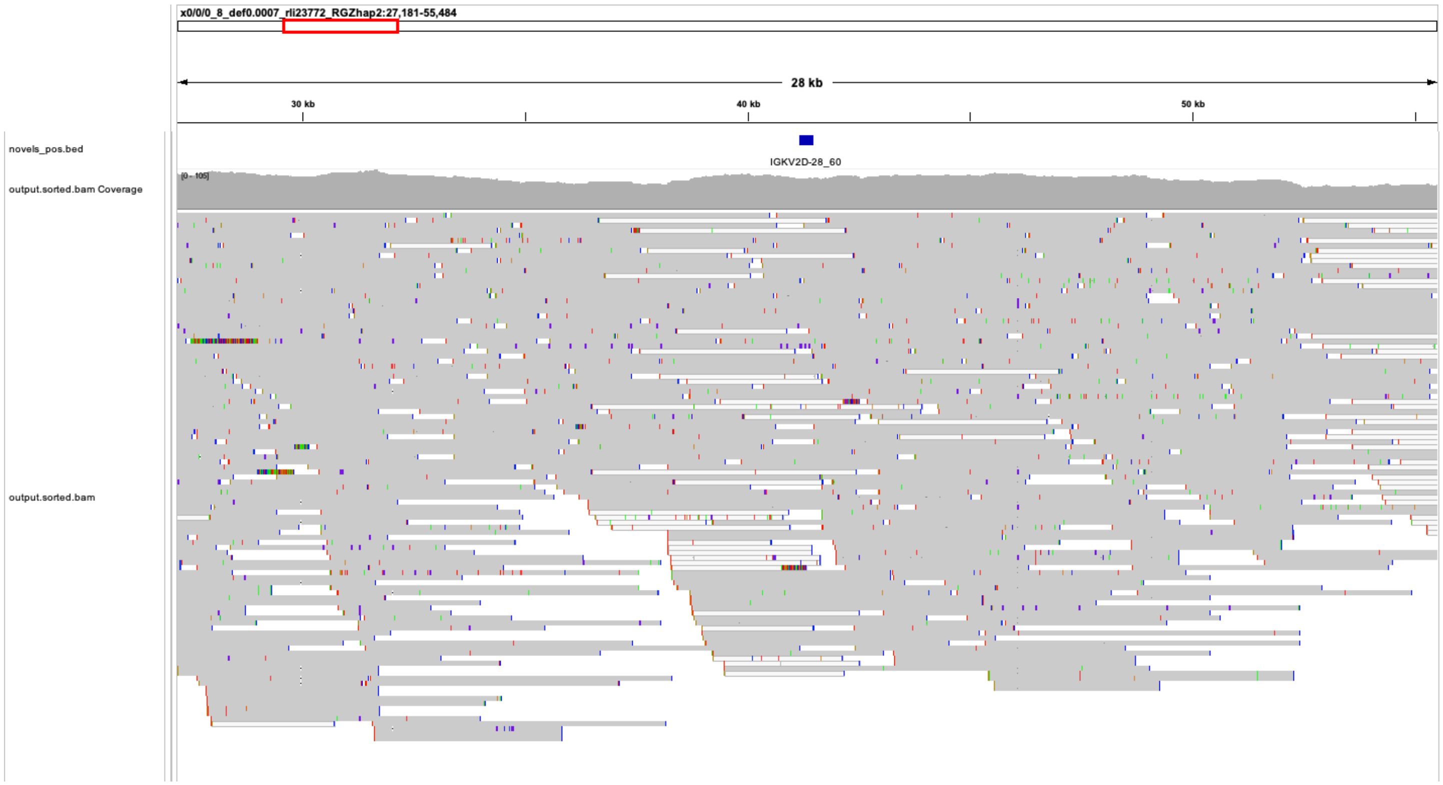


Sample: HG01887

# Figure S9
(continued)

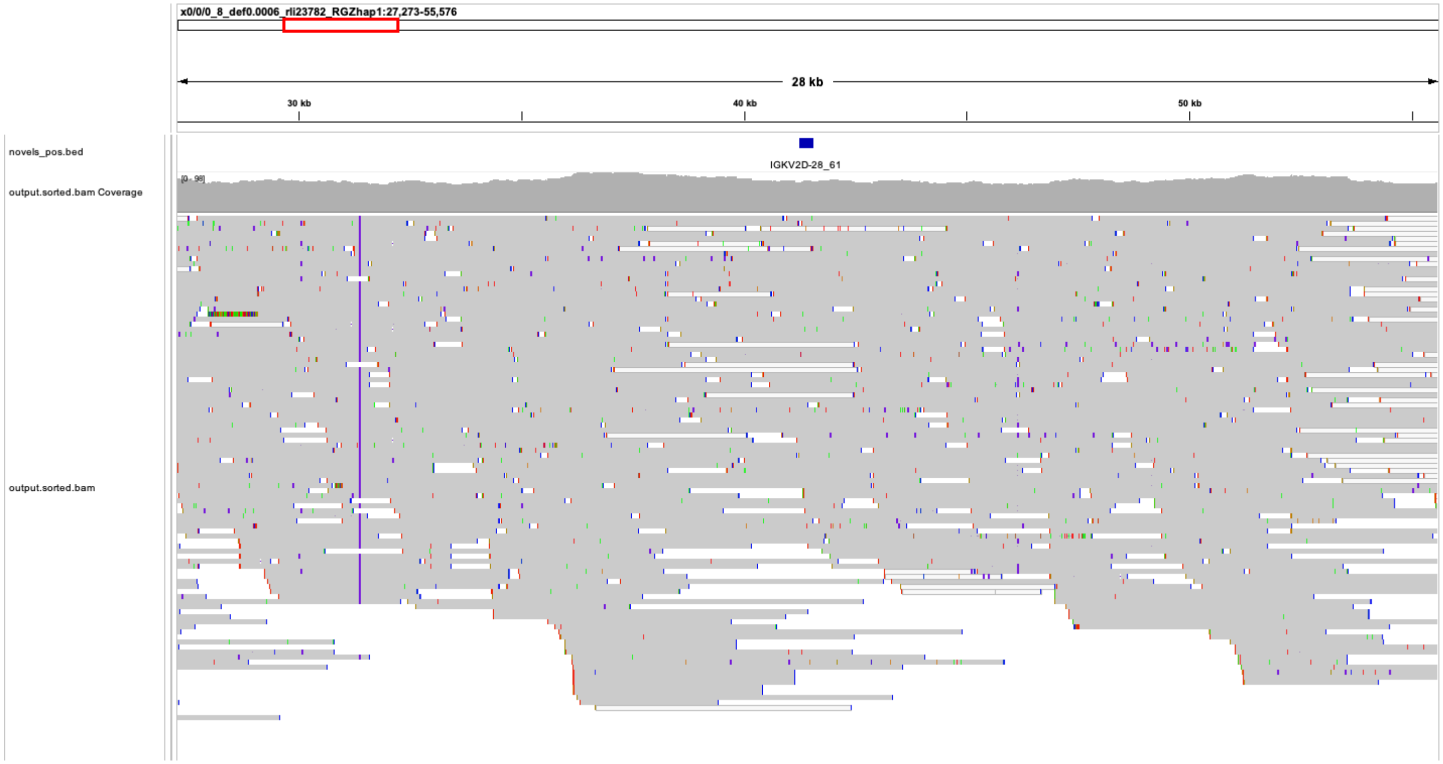


Sample: HG01887

# Figure S9
(continued)

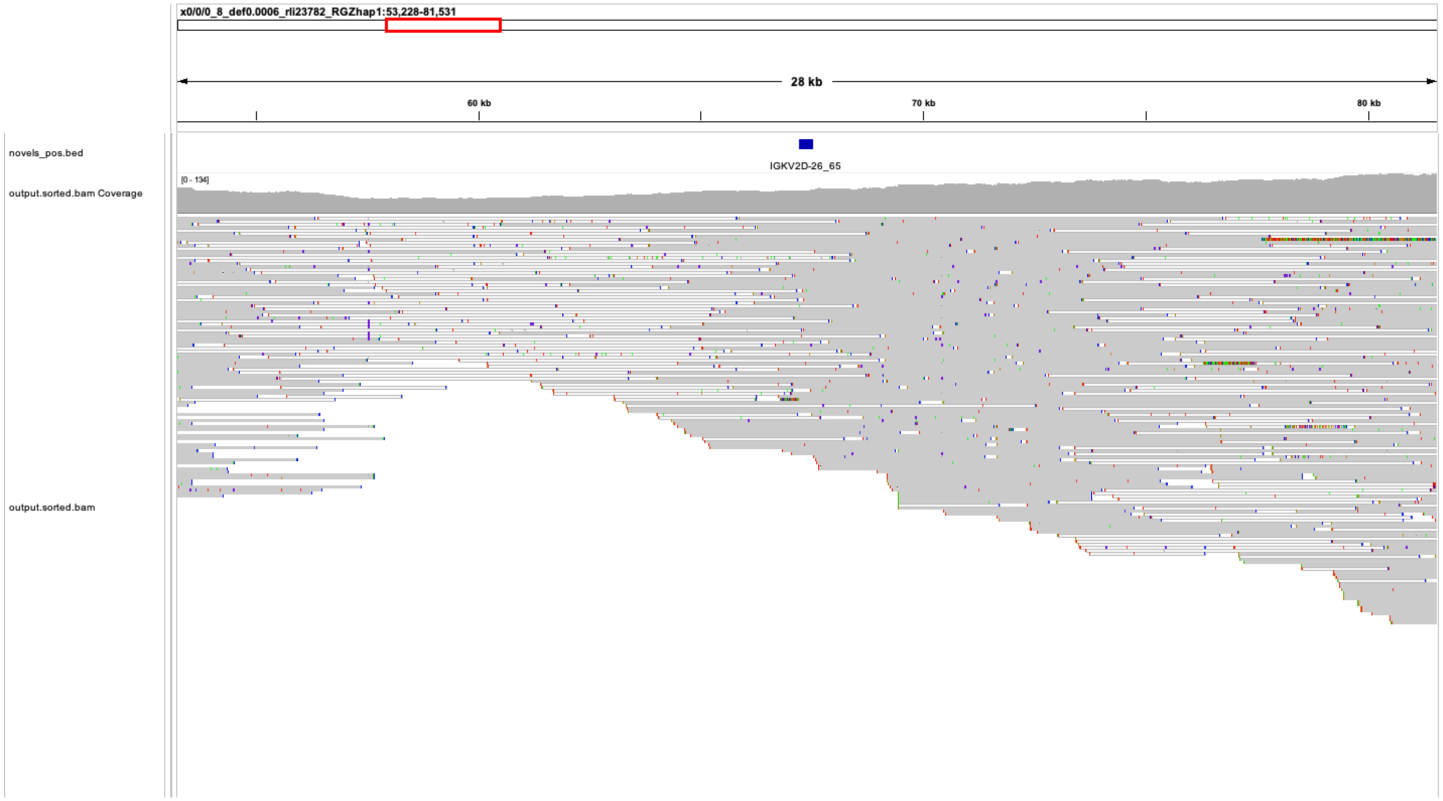


Sample: HG01887

Figure S9
(continued)

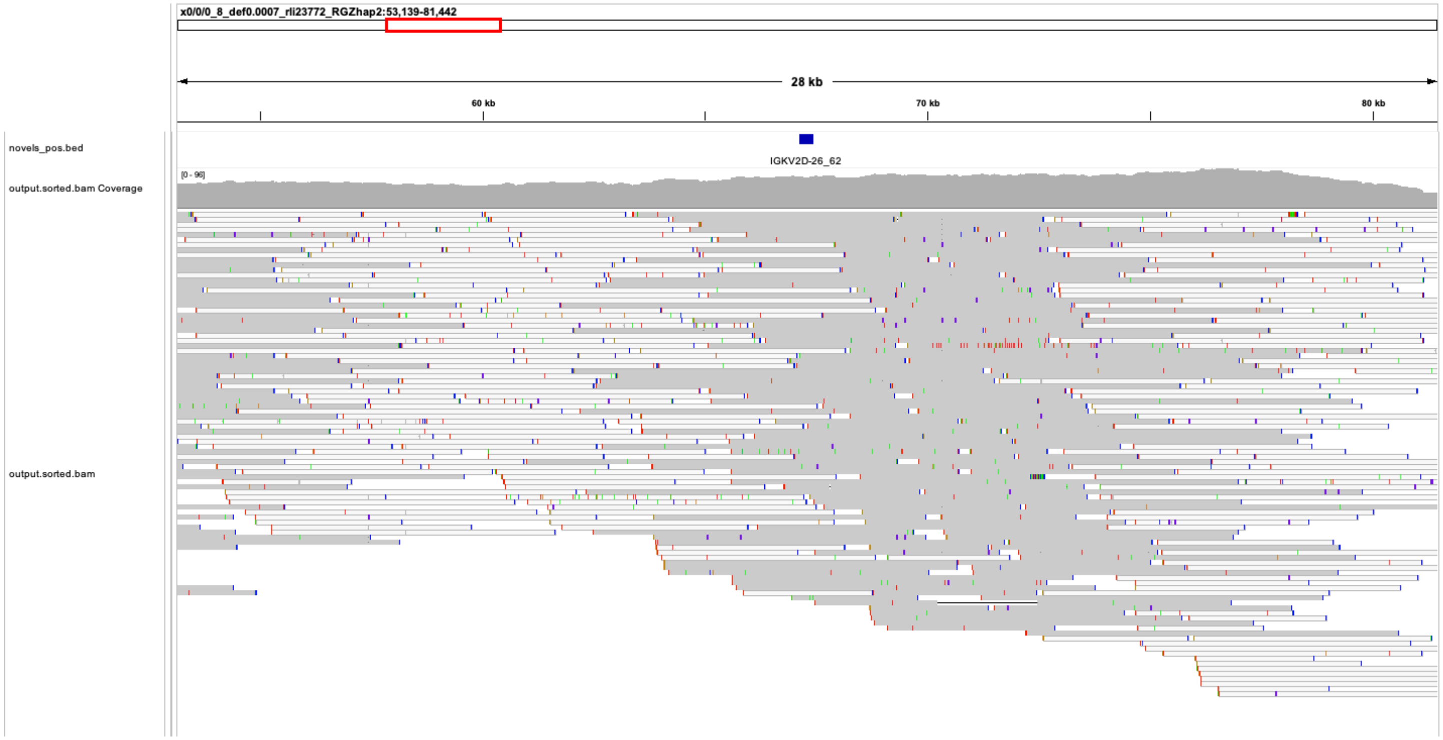


Sample: HG01887

Figure S9
(continued)

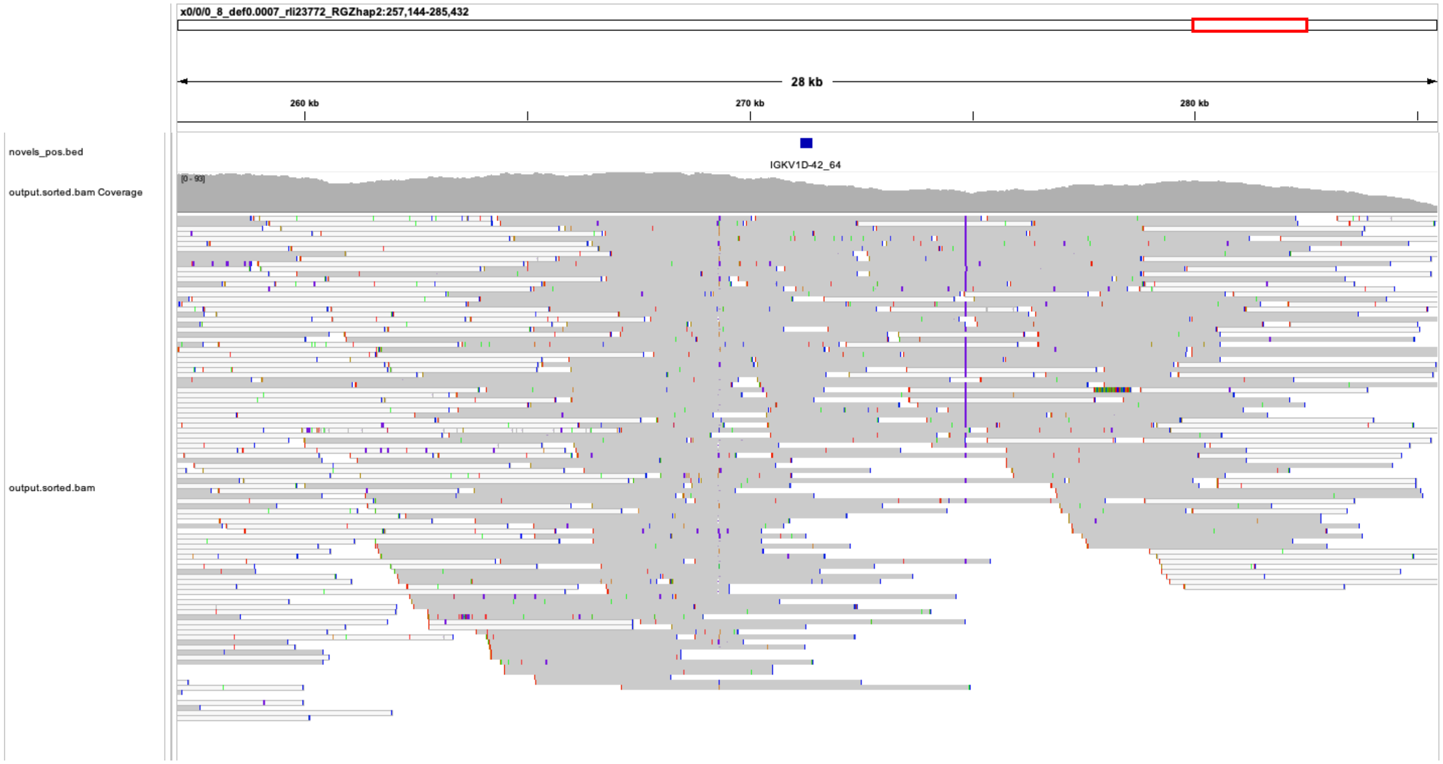


Sample: HG01887

# Figure S10

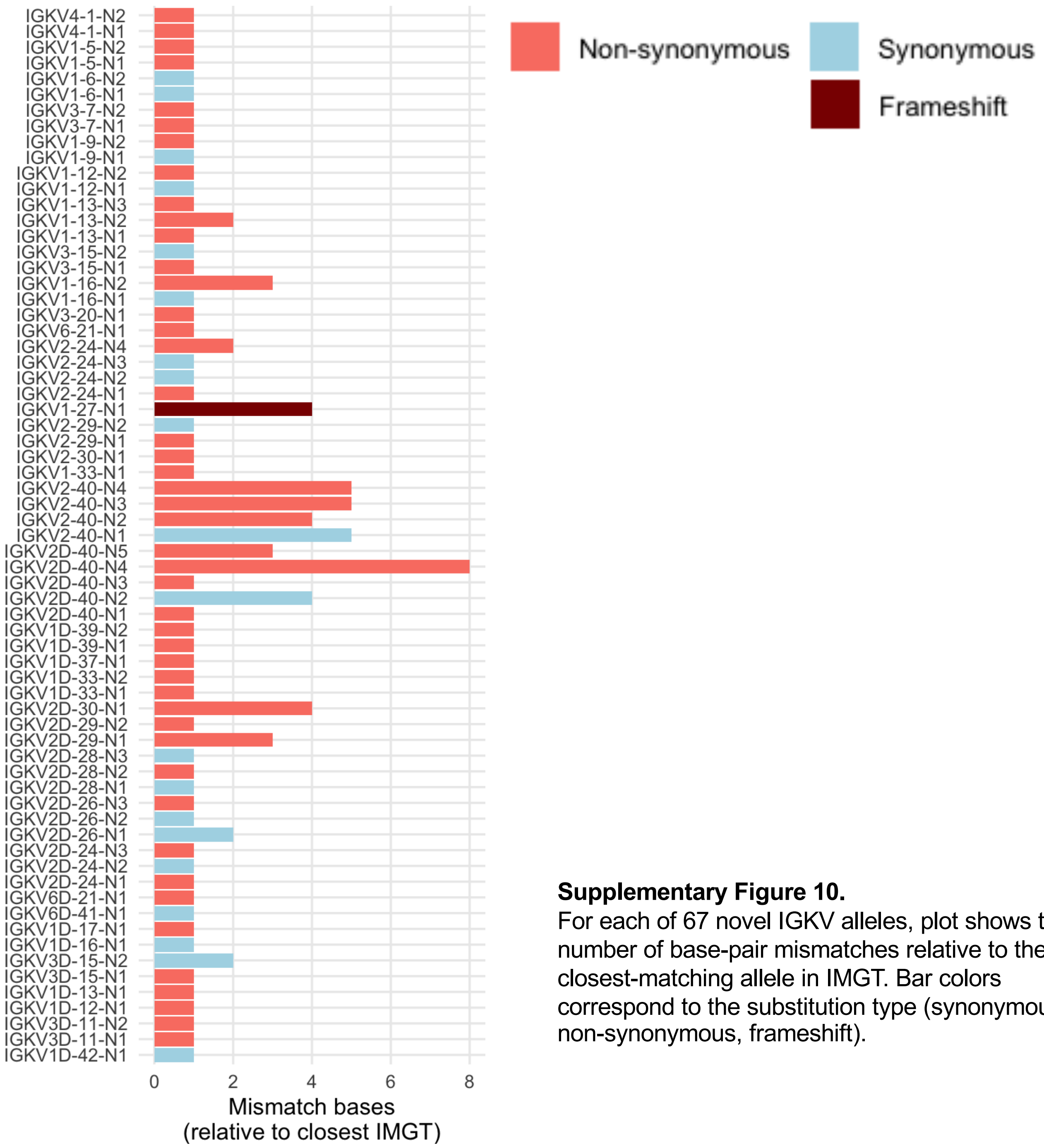


**Supplementary Figure 10.**
For each of 67 novel IGKV alleles, plot shows the number of base-pair mismatches relative to the closest-matching allele in IMGT. Bar colors correspond to the substitution type (synonymous, non-synonymous, frameshift).

Figure S11

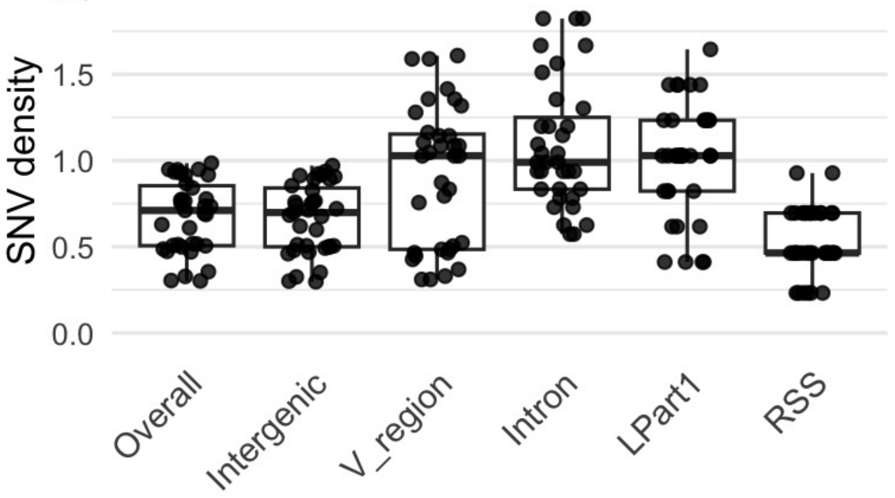


| Comparison | Padj (Tukey's post-hoc) |
|---|---|
| V_region-Overall | 0.014818171 |
| Intron-Overall | 4.00E-07 |
| LPart1-Overall | 6.61E-05 |
| V_region-Intergenic | 0.008374841 |
| Intron-Intergenic | 1.62E-07 |
| LPart1-Intergenic | 3.03E-05 |
| RSS-V_region | 3.66E-06 |
| RSS-Intron | 3.79E-12 |
| RSS-LPart1 | 2.10E-09 |

**Supplementary Figure 11.**
SNV densities for indicated genomic features for 35 samples (data points). Table indicates adjusted P-values from Tukey's post-hoc test. Comparisons not shown have adjusted P-values > 0.05.

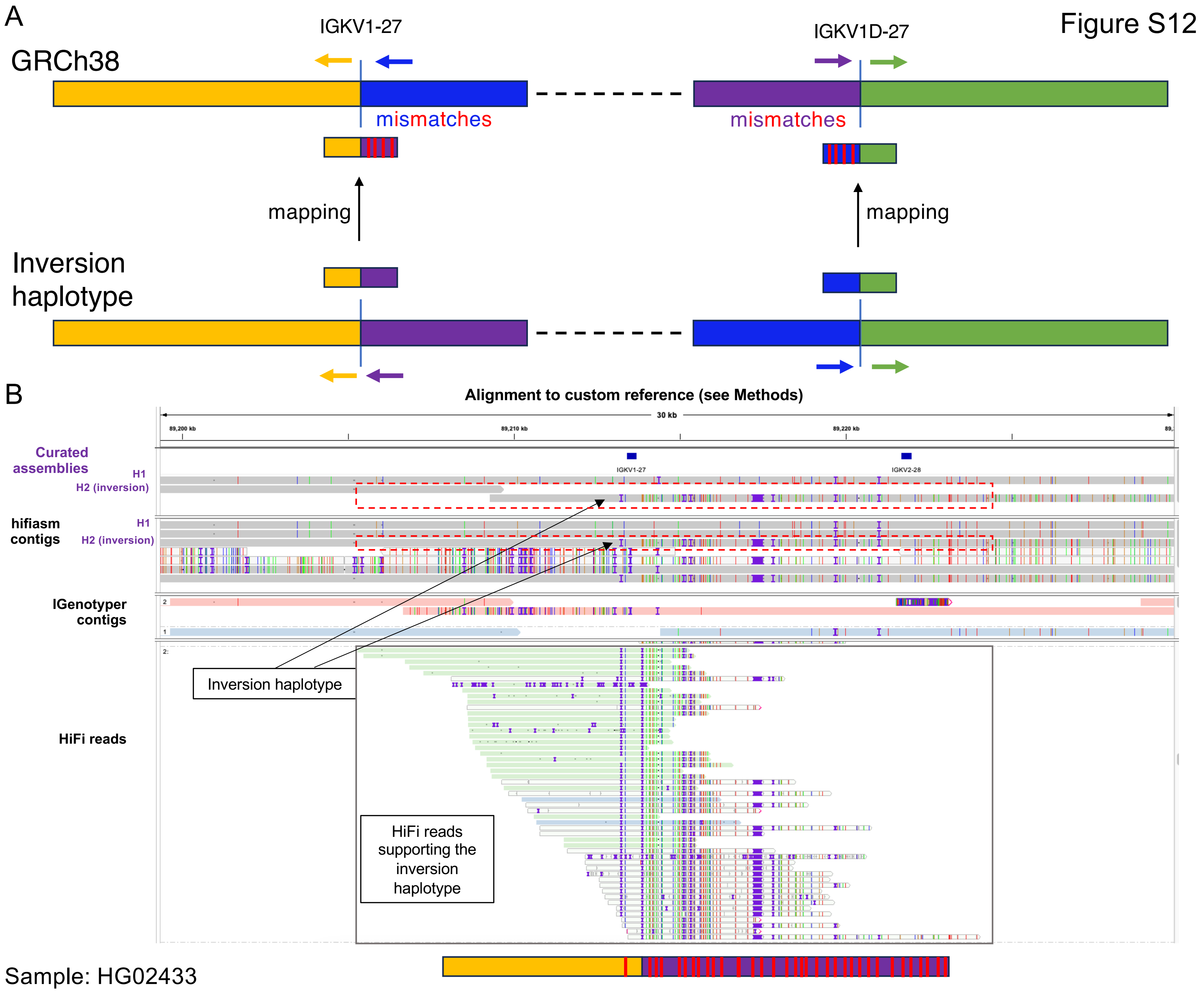

Figure S12
A
GRCh38
IGKV1-27
IGKV1D-27
mismatches
mismatches
mapping
mapping
Inversion haplotype
B
Alignment to custom reference (see Methods)
30 kb
89,200 kb
89,210 kb
89,220 kb
Curated assemblies
H1
H2 (inversion)
IGKV1-27
IGKV2-28
hifiasm contigs
H1
H2 (inversion)
IGenotyper contigs
HiFi reads
Inversion haplotype
HiFi reads supporting the inversion haplotype
Sample: HG02433

Figure S112
(continued)

**Supplementary Figure 12.**
**(A)** Schematic of the GRCh38 assembly and inversion haplotype identified in HG02433.
Shown is a schematic of a read that spans the inversion breakpoints at the IGK proximal gene IGKV1-27 and the IGK distal pseudogene IGKV1D-27. Sequence towards the gap extending from each breakpoint (blue and purple regions) are inverted on one haplotype of HG02433 relative to the GRCh38 assembly. **(B-C)** Shown are curated assemblies (H1 and H2), hifiasm contigs, IGenotyper contigs, and HiFi reads aligned to our custom GRCh38 reference. White-shaded contig mappings are secondary alignments. Hifiasm-generated contigs representing the inversion haplotype are indicated with arrows; HiFi reads that span the inversion breakpoints and support these hifiasm contigs are boxed. The contig spanning the IGKV1-27 breakpoint region mapped as a primary alignment, whereas the contig spanning the IGKV1D-27 breakpoint mapped as a secondary alignment.

C

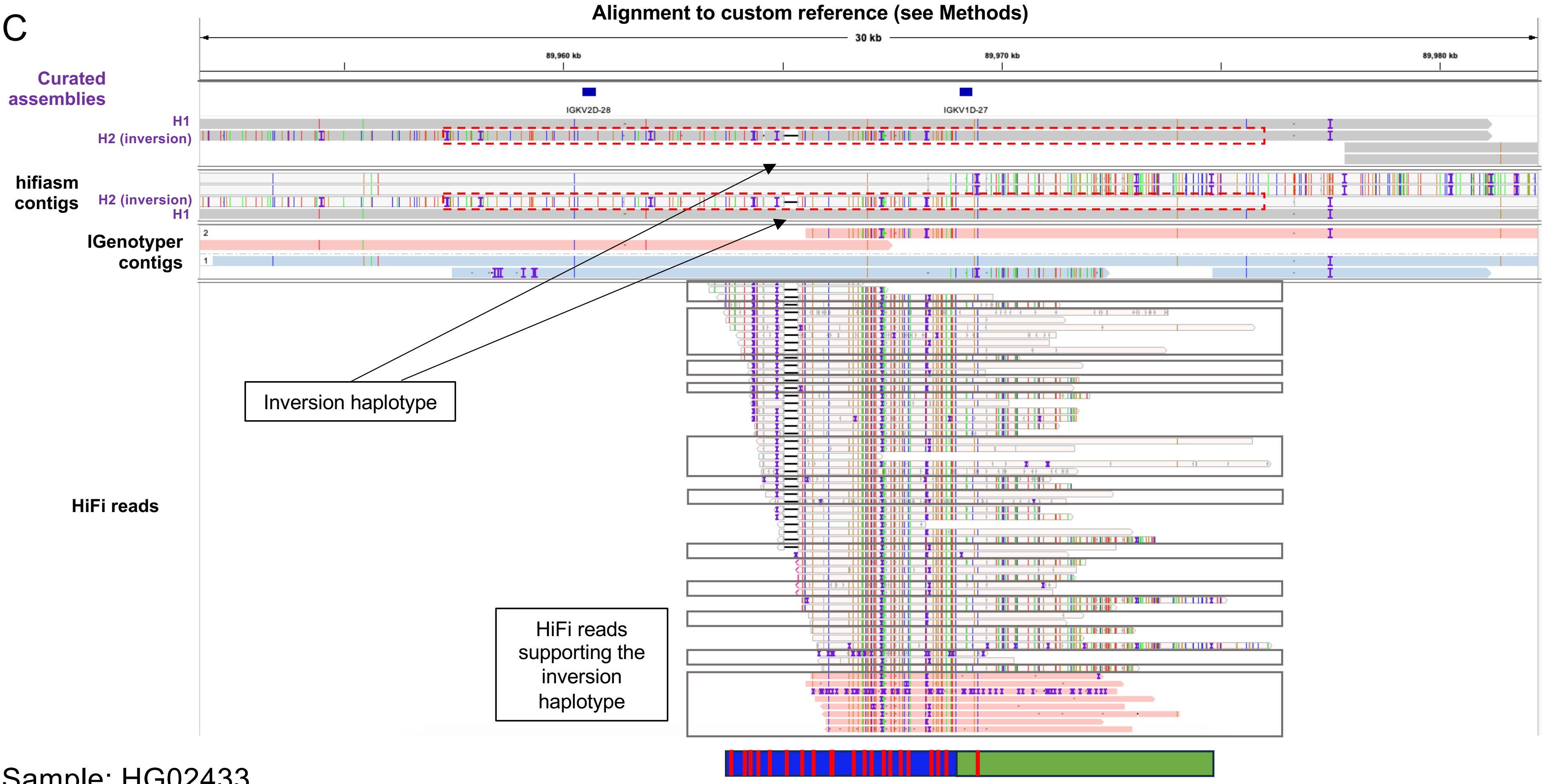


Sample: HG02433

# Figure S13

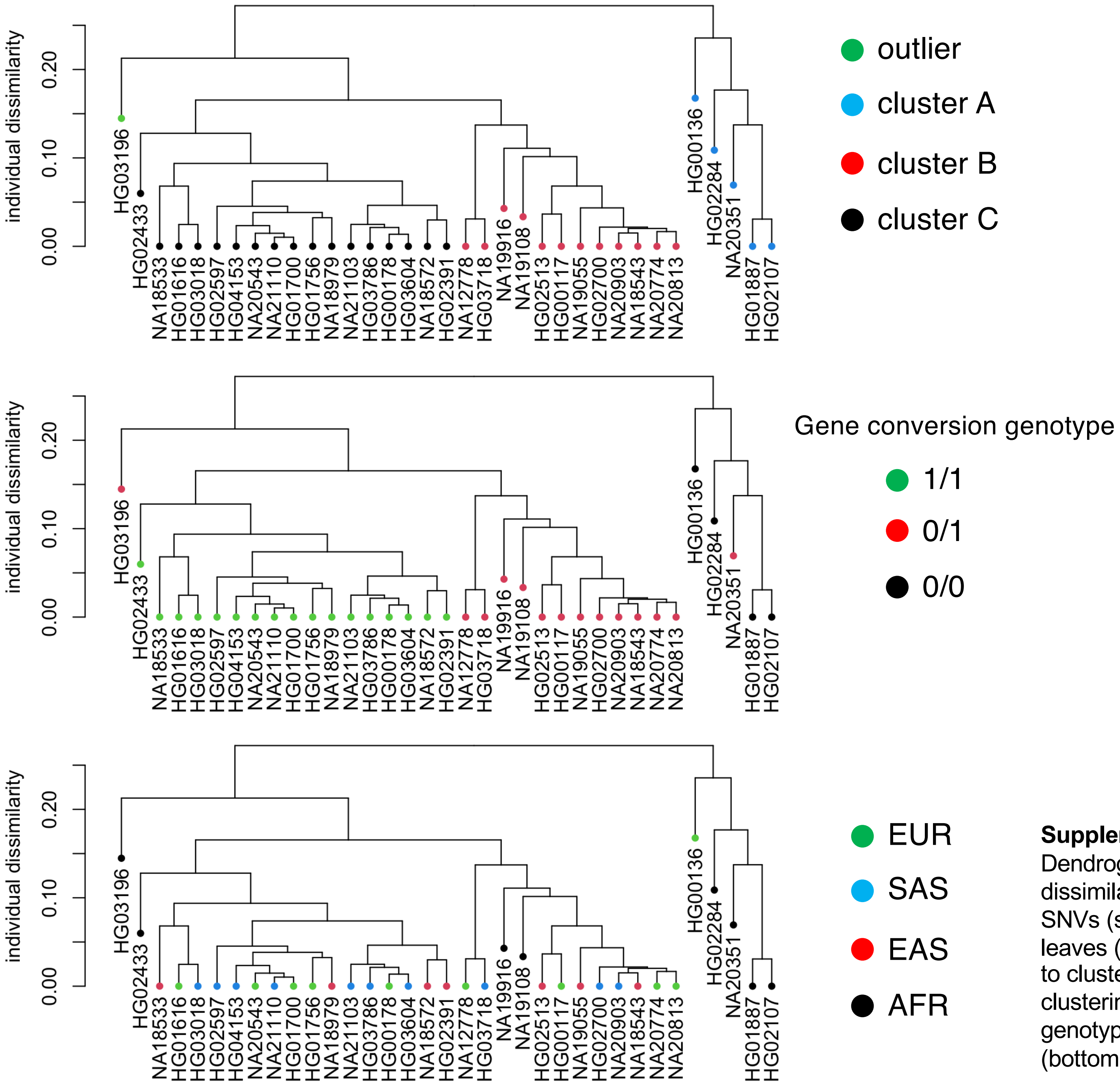


**Supplementary Figure 13.** Dendrogram resulting from a pairwise dissimilarity matrix of distal region SNVs (see **Methods**). Dendrogram leaves (samples) are colored according to cluster resulting from hierarchical clustering (top), gene conversion genotype (middle), or population (bottom).

# Figure S14

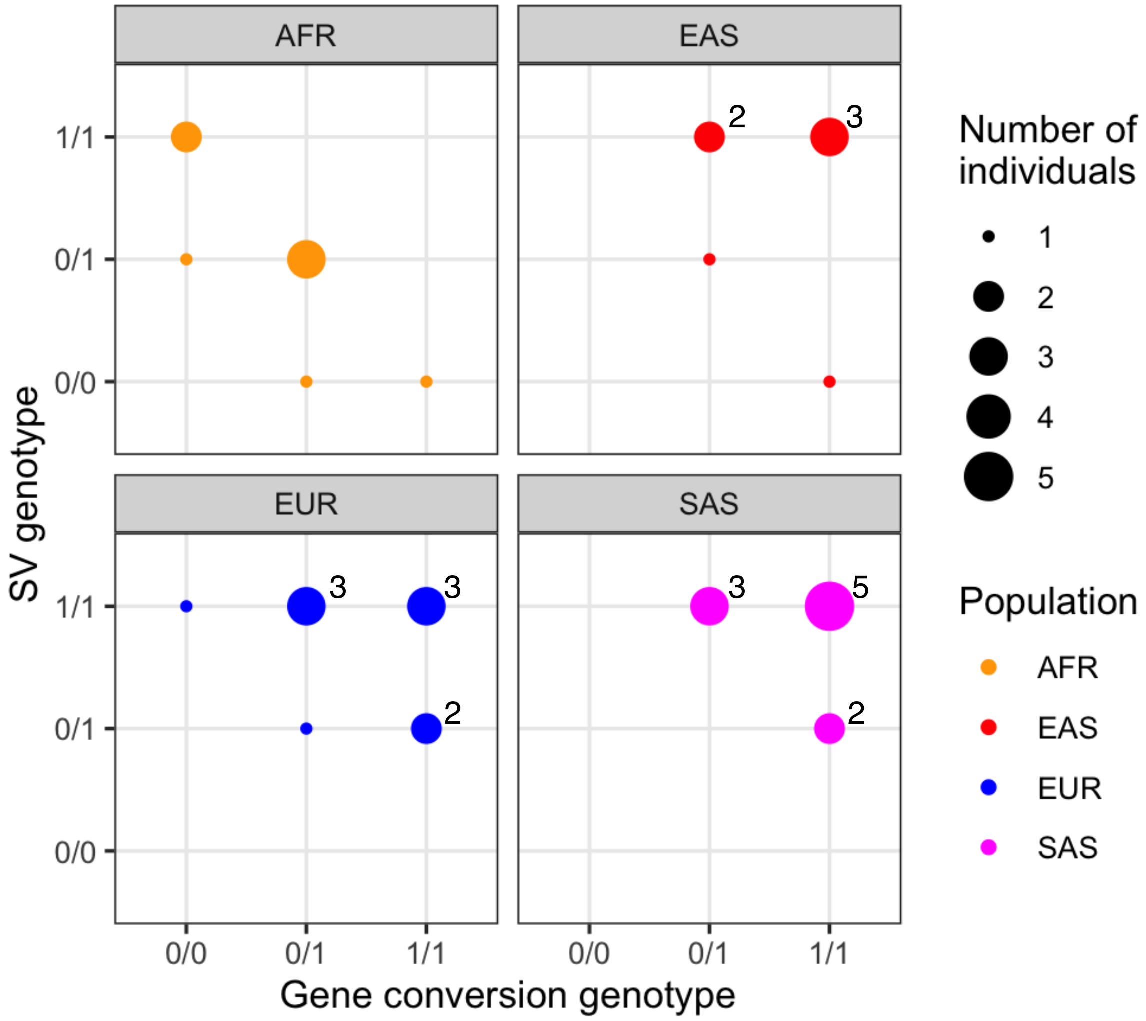


**Supplementary Figure 14.**
Bubble plot showing the number of samples from different populations with each combination of genotypes for the gene conversion and the structural variant that includes IGKV1-NL1.

# Figure S15

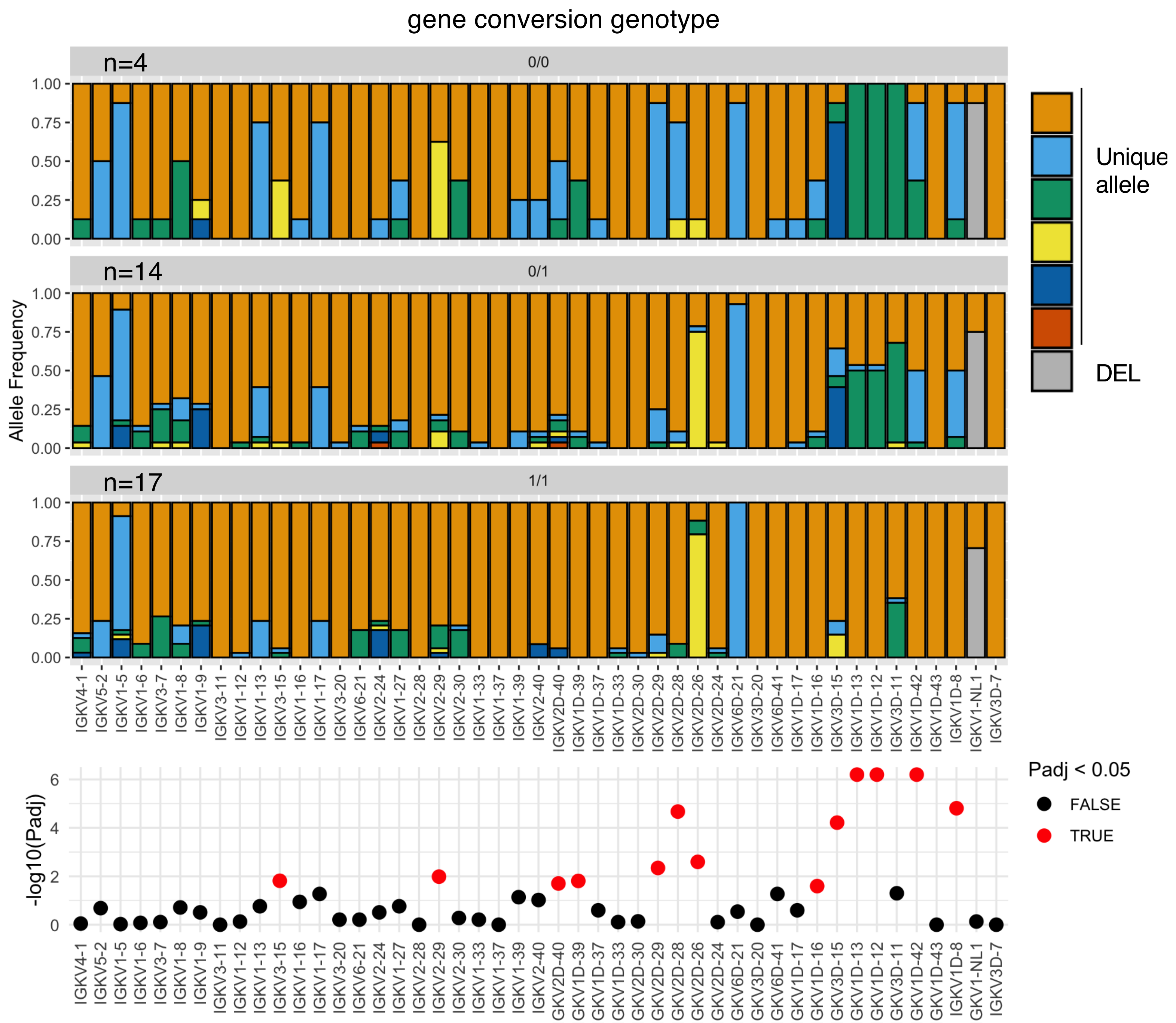


**Supplementary Figure 15.** **(A)** Stacked bar plot of allele frequencies for each of 47 functional IGKV genes for individuals stratified by genotype for the gene conversion. For each gene, a color corresponds to a unique allele. The *02 alleles for IGKV1D-12 and IGKV1D-13 are represented by green color. Gray color indicates allele absence due to structural variation (deletion). **(B)** Differences in allele frequency distributions among genotype groups were determined using a chi-square test; Benjamini-Hochberg adjusted P-values (Padj) are plotted as -log10(Padj) for each gene. Red points indicate Padj < 0.05.

# Figure S16

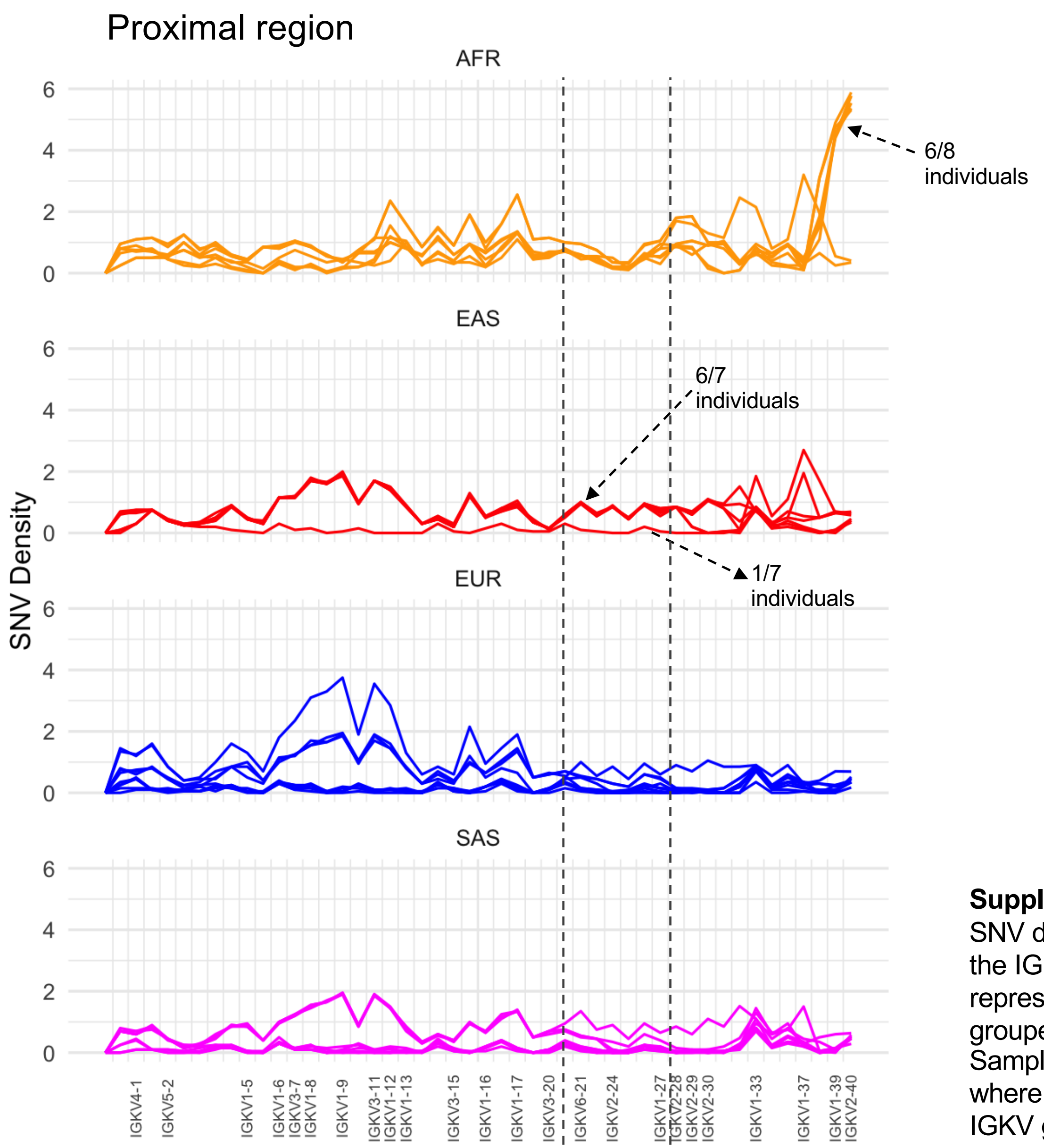


**Supplementary Figure 16.**
SNV densities in 10 Kbp windows along the IGK proximal region. Each line represents an individual. Individuals are grouped according to population. Samples are indicated in selected regions where lines (samples) are overlapping. IGKV gene positions are indicated.

Figure S17

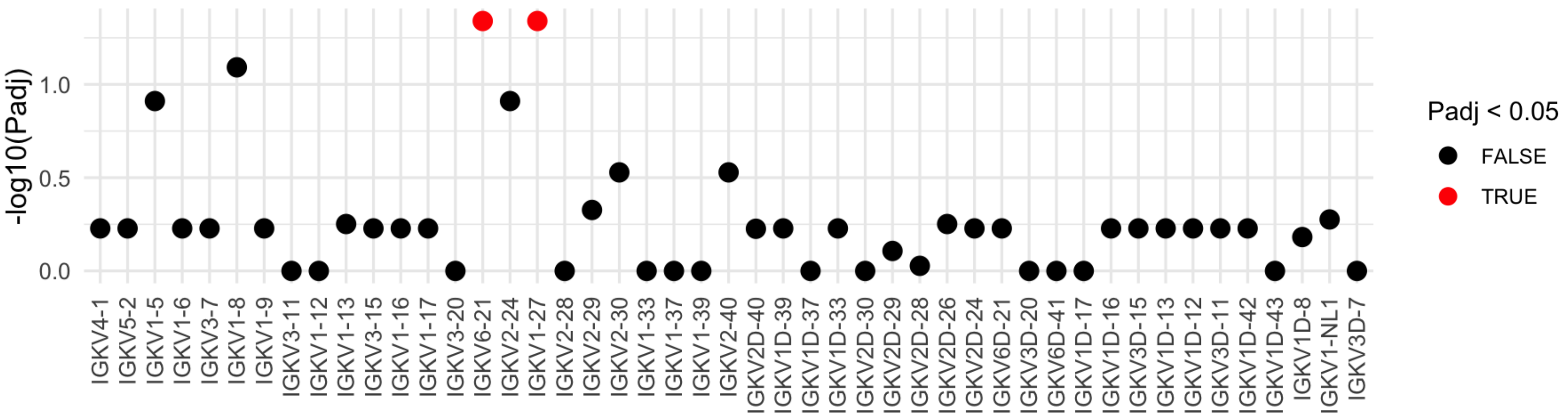


**Supplementary Figure 17.**
Differences in allele frequency distributions among population groups with individuals of AFR ancestry removed. P-values were determined using a chi-square test; Benjamini-Hochberg adjusted P-values (Padj) are plotted as -log10(Padj) for each gene. Red points indicate Padj < 0.05.
